# An Investigation of Dynamic Responses and Head Injuries of Standing Subway Passengers during Collisions

**DOI:** 10.1155/2018/1096056

**Published:** 2018-09-02

**Authors:** Yong Peng, Tuo Xu, Lin Hou, Chaojie Fan, Wei Zhou

**Affiliations:** ^1^Key Laboratory of Traffic Safety on Track (Central South University), Ministry of Education, School of Traffic & Transportation Engineering, Central South University, Changsha 410075, China; ^2^State Key Laboratory of High Performance Complex Manufacturing, Central South University, Changsha 410006, China; ^3^Joint International Research Laboratory of Key Technology for Rail Traffic Safety, Central South University, Changsha 410075, China; ^4^National & Local Joint Engineering Research Center of Safety Technology for Rail Vehicle, Central South University, Changsha 410075, China

## Abstract

With the development of the subway and the pressing demand of environmentally friendly transportation, more and more people travel by subway. In recent decades, the issues about passenger passive safety on the train have received extensive attention. In this research, the head injury of a standing passenger in the subway is investigated. Three MADYMO models of the different standing passenger postures, defined as baseline scenarios, are numerically set up. HIC_15_ values of passengers with different postures are gained by systematic parametric studies. The injury numerical simulation results of various scenarios with different friction coefficients, collision acceleration, standing angle, horizontal handrail height, and ring handrail height are analyzed. Results show that the horizontal handrail provides better protection in the three different standing passenger postures. Different friction coefficients and the standing angle have great impact on the head injuries of passengers in three different scenarios. The handrail height also has some effects on head injury of passengers with different standing postures, so it is necessary to be considered when designing the interior layout of the subway. This study may provide guidance for the safety design of the subway and some advices for standing subway passengers.

## 1. Introduction

The subway has become one of the most important travel modes for urban residents, owing to its convenience. Due to the characteristics of high speed, high quality, and high passenger density, subway vehicles will cause unbearable casualties in the subway collision accident. In 2009, the collision accidents of subway occurred in Washington, USA, causing nearly one hundred casualties [1]. In 2011, a subway collided with a truck, causing many casualties in Los Angeles, USA [2].

In general, many countries have focused on the crashworthiness of rail vehicle in the 80s of last century. In the United Kingdom, Railway Safety and Standards Committee (RSSC) revised the GM/RT 2100Iss4. It contained detailed requirements of the interior of the train, such as interior trim and seat spacing. Subsequently, the UK put forward the crashworthiness standards AV/ST 9001 and started to research how to reduce the occupant secondary collision injury by changing the interior design.

In the United States, there have been multiple studies addressing rail crashworthiness and occupant safety [3–7]. The Federal Railroad Administration (FRA) sponsored studies on the vehicle collision response and crashworthiness performance of vehicles by computer analysis and experiments [4–6]. The US Volpe National Transportation Systems Center carried out a series of real-vehicle crash tests and occupant secondary collision tests [4]. They made some measures to reduce occupant secondary collision injury [5]. Simons and Kirkpatrick used the vehicle crash response to calculate the acceleration environment of the car interior in mathematical simulation. Separately, they applied this acceleration environment to simulate the response and injury of passengers in seated configurations [6]. On bus collisions, vehicle compatibility issues were proposed during typical mass transit bus collisions with sedans, light trucks, and heavy trucks through the use of numerical finite element simulations [7].

In Japan, as early as 1997, the casualties of passengers in train collisions were brought into sharp focus [8, 9]. In 2002, the spontaneous self-protection posture was proposed in the research of passenger injury [10]. Subsequently, in 2008, the passengers' behavior on benches in train collisions was studied [11]. In 2012, several researches studied the behavior of commuter trains in the crossing accidents. The authors found that armrests or baffles could reduce the possibility of passenger damage [12].

In the European Union, SAFETRAIN, SAFETRAM, TRAINSAFE, SAFE INTERIORS, and other projects carried out related research on the crashworthiness and occupant safety of urban rail vehicles. These included studies of sitting posture, occupant injury in the interior environment, and the crash energy management (CEM) requirements of different vehicles. These efforts supported the development of the European railway crashworthiness standard EN15227 in 2007.

In China, there were some crashworthiness researches in universities. A model of a console-seat-dummy which optimizes the driver's workspace was proposed [13]. Subsequently, the main influencing factors of the passenger injury [14] and the energy absorption requirements of the crash vehicle [15] were considered. The requirements were a great help to vehicle structure design. Later, the dynamic response of occupant secondary collision [16] aimed to forecast impact injuries.

Current study focuses on related issues of train collision safety. The research found that majority of the injuries to occupants are a result of secondary collisions between occupants and other objects [17–19]. The occupant secondary collision means that the injury is caused by the contact with the interior of the vehicle [20–22]. The head injury is the most investigated traffic injury, and the head injury criterion (HIC) is the most commonly used injury index to passengers [20–25]. However, the safety issues of subway passengers are ignored, due to the characteristics of subway vehicles, including the high speed, high quality, and high passenger density. To bridge the gap, the objective of our study is to assess head injury risks of standing subway passengers and therefore to give some advices on preventing serious injuries of these passengers. In Section 2, accident scenarios, including human and vehicle models and finite element (FE) head-ground impact model, are established. In Section 3, the injury results are analyzed under three baseline scenarios. In Section 4, comprehensive parametric discussions are presented to indicate the mechanism of head injuries of standing subway passengers.

## 2. Methods

To assess the head injuries of standing passengers during a crash, three different standing passenger postures, that is, horizontal handrail passenger, ring handrail passenger, and vertical handrail passenger, are considered. Almost all standing subway passenger scenes are covered. Numerical simulation scenes are set up based on MADYMO (MADYMO 7.5, Netherlands Organisation for Applied Science Research, Delft, Netherlands) [26] platform. The MADYMO platform is used to study the vehicle crash safety [27].

**Figure 1:**
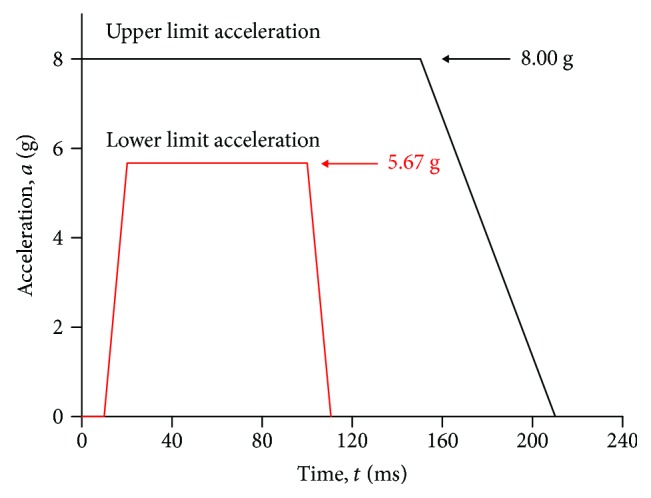
Collision acceleration curve as defined in AV/ST 9001 vehicle interior crashworthiness.

In the analyses, the impact condition specified in EN15227 standard is applied (shown in Figure 1) [28]. The wet weather conditions in this area and subway model referred are taken into consideration. Therefore, the baseline scenario of three different standing passenger postures is set with lower limit acceleration in 5.67 g (shown in Figure 1), the static coefficient friction between shoes and floor with 0.49 [29], and standing angle with 0°, and the heights of the horizontal handrail and the ring handrail are 1850 mm and 2000 mm, respectively.

**Figure 2:**
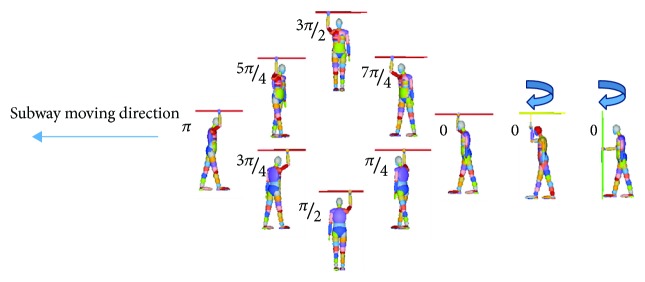
Description of different standing angles.

To further quantify the collision scenarios, numerical simulations are conducted at various collision acceleration, coefficient friction (between foot and floor), standing angle, and handrail height (from handrail to floor) with a total of 270 numerical simulations (shown in Table 1). Numerical collision conditions are simulated with the collision acceleration changed every 0.5 g range from 2 g to 10 g. The different gradients of the coefficient friction are set from 0.49 (baseline scenarios) to 0.85 with the interval of 0.05. The standing angle is set as dominant variables, that is, 0, *π*/4, *π*/2, 3*π*/4, *π*, 5*π*/4, 3*π*/2, and 7*π*/4 (shown in Figure 2). The heights of the horizontal handrail and the ring handrail are set from 1830 mm to 1950 mm and 1950 mm to 2050 mm with the interval of 10 mm, respectively.

In consideration of the head injury importance, we take finite element head models to analysis by simulating boundary conditions in the baseline scenario, individually. The results of MADYMO simulations for the baseline scenario with three different standing passenger postures are used as the boundary conditions for finite element simulations later. The head impact boundary conditions include linear velocity, angular velocity, the head position, and head linear acceleration [30].

### 2.1. Accident Scenarios

**Figure 3:**
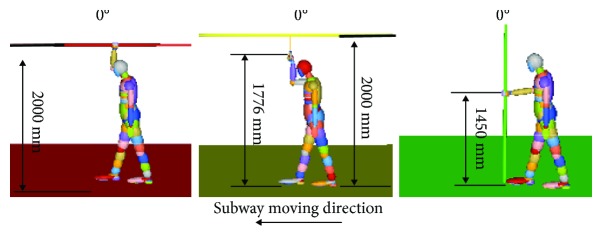
The standing models and standing angle to describe horizontal handrail, ring handrail, and vertical handrail.

Three standing models in the baseline scenario are shown in Figure 3. The relevant design parameters of the subway handrails correspond to actual designs in service in China.

### 2.2. Human Model

The 50th percentile male pedestrian model (1.74 m, 75.7 kg) incorporated within MADYMO [31] is chosen to represent the standing subway passenger. This model is widely accepted for accident analysis and reconstruction studies to assess human body kinematics and injury potential [32, 33]. The pedestrian model consists of 52 rigid bodies, with an outer surface described by 69 ellipsoids, and there are 52 joints within the human model.

### 2.3. Hand Model

Between the hand model and the handrail, we define a contact with failure to simulate the hand grip force [29]. The average grip strength for males aged 20–59 is 450 N in China [34]. When the hand grip force reaches 450 N, the contact between the hand model and the handrail becomes invalid.

### 2.4. Injury Evaluation Index

HIC is widely accepted for assessing the severity of head injuries [9, 10, 23, 30, 35, 36]. HIC_15_ is well correlated with averaged angular acceleration [9] and can avoid some of the potential errors [10]. Consequently, HIC_15_ is chosen during the secondary impact.

### 2.5. Finite Element (FE) Head-Ground Impact Model

**Figure 4:**
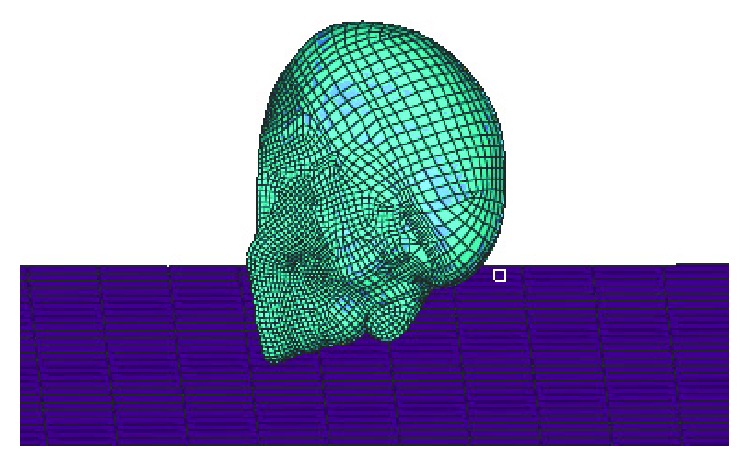
The FE head-ground impact model.

The HBM-head model is adopted to build the FE head-ground model [36, 37]. The HBM-head model has been validated and widely used in the field of skull and brain injury research [38–42]. The subway aluminum honeycomb ground FE model is constructed [43].  Figure 4 shows the FE head-ground impact model. The head-ground impact process is reconstructed by using three coordinate points to get the relative position of the HBM-head with ground. The linear velocities, angular velocities, and linear acceleration are used as the boundary condition. This method has been adopted to study the influence of head mass on temporoparietal skull impact [44].

## 3. Results

**Figure 5:**
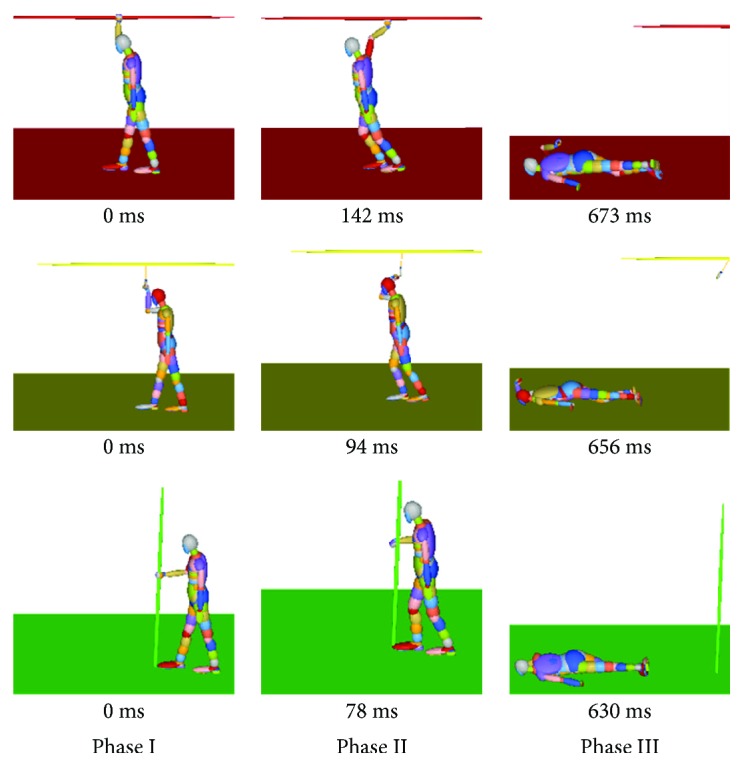
Dynamic responses of three passengers with different standing postures in the baseline scenario.

From the dynamic point of view, the standing subway passenger accident could be divided into three phases, that is, hand-handrail contact phase (I), hand-handrail separation phase (II), and head-floor contact phase (III), indicated in Figure 5. In phase I, three kinds of standing subway passengers hold the handrail in three different ways. In phase II, the hand grip force of the passenger reaches 450 N. The contact between the hand model and the handrail becomes invalid. In phase III, the standing subway passenger falls down and the head and floor make contact. The kinematic mechanisms of the standing subway passenger in three postures are demonstrated by MADYMO.

### 3.1. The Resultant Hand Grip Force

**Figure 6:**
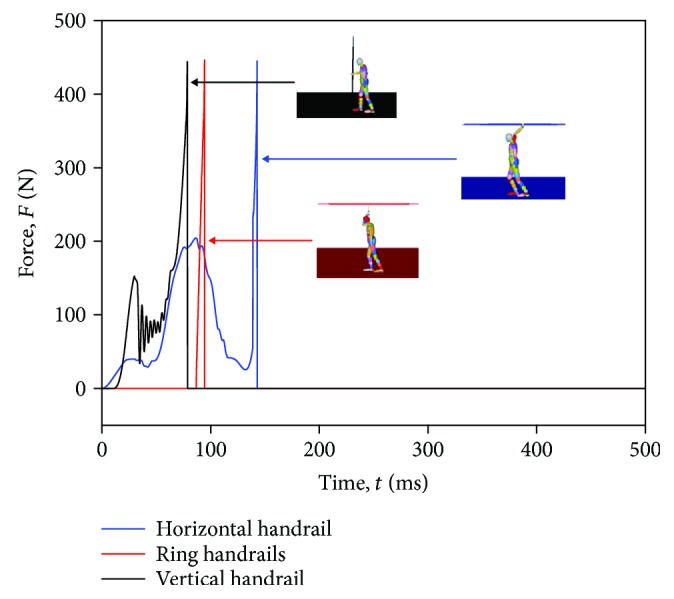
Resultant head-handrail forces of three different standing passenger postures in the baseline scenario.

**Figure 7:**
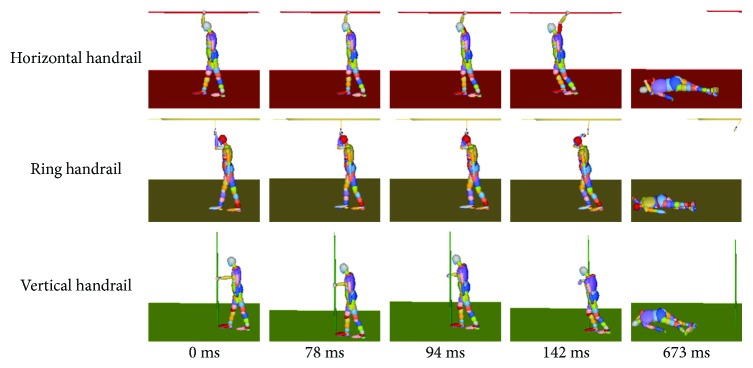
The dynamic behaviors to the hand grip force.

The hand grip force reflects directly the time when the hand and the handrail separate. As shown in Figure 6, the hand grip force of the horizontal handrail passenger, the ring handrail passenger, and the vertical handrail passenger reaches 450 N in 142 ms, 94 ms, and 78 ms, respectively. The horizontal handrail has the greatest effect on the overall passenger response (integrating the force-time history), and the ring handrail has the least effect. It can be observed in the behaviors (Figure 7). For the horizontal handrail, the hand postures of a standing passenger change from 0 ms to 142 ms, while it keeps the same from 0 ms to 94 ms for the ring handrail. There are some fluctuations in the horizontal handrail curve and the vertical handrail curve before reaching the peak. It can be explained that the hand does not touch the ring handrail in the beginning.

### 3.2. The Resultant Head Acceleration

**Figure 8:**
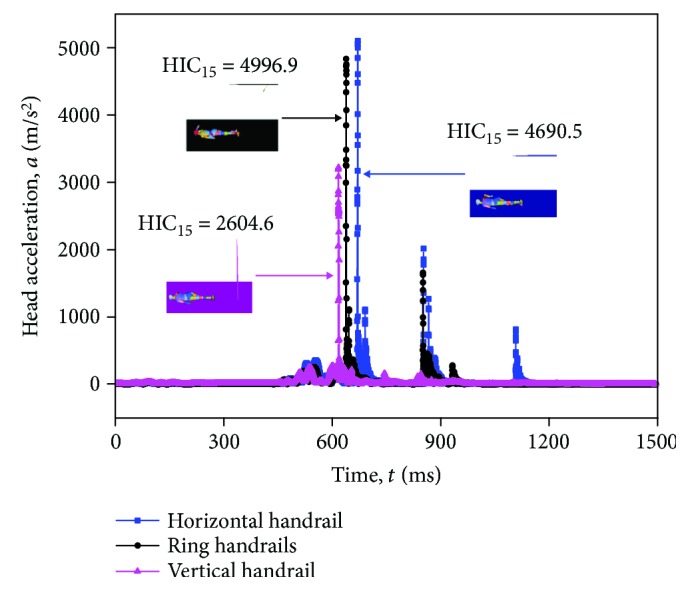
Resultant head accelerations of three passengers with different standing postures in the baseline scenario.

For the same impact scenarios selected in the previous section, three baseline scenarios in three postures present the representative simulation results. As shown in Figure 8, for the horizontal handrail and the ring handrail during the head-floor contact, there are several small peaks in head acceleration curve, while a peak in the vertical handrail. The reason is that the arm plays a buffer role when the standing subway passenger in the vertical handrail falls down. The head acceleration reaches the peak when the head and floor make contact for the first time. The peak in the horizontal handrail and ring handrail arrives late, compared with that in the vertical handrail. The peak value maximum in the vertical handrail is the smallest in three standing postures, that is, HIC_15_ = 2604.6. It can be explained that the arm-floor contact mitigates a part of impact energy. For the standing subway passenger in the ring handrail, the time for head-floor contact is longer than the time in the horizontal handrail. This leads to the greater value of HIC_15_, that is HIC_15_ = 4996.9 and HIC_15_ = 2604.6, respectively.

### 3.3. The Head Center of Gravity (CG) Displacement

**Figure 9:**
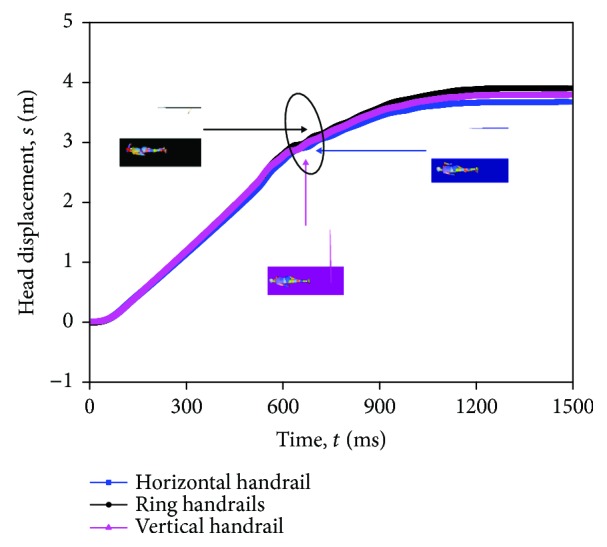
Head CG displacement-time curves of three passenger standing postures in the baseline scenario.

The displacement of the head center of gravity (CG) reflects directly the kinematic movement of the standing subway passenger in three standing postures. The head displacement is the horizontal displacement along the collision direction of the car body from falling over. The displacement is relative to a reference frame defined by a fixed position on the floor on the car body. The head displacement increases almost linearly at first. This indicates that the head velocity keeps constant. An abrupt gradient change can be observed in the displacement-time history curve in the three standing postures (Figure 9). The constraint of head movement is the head-floor contact. The gradient happens at about 620 ms, and the three postures of the standing subway passenger head contact the floor, that is, 634.6 ms, 621.5 ms, and 618.6 ms, respectively.

### 3.4. The Head Center of Gravity (CG) Speed

**Figure 10:**
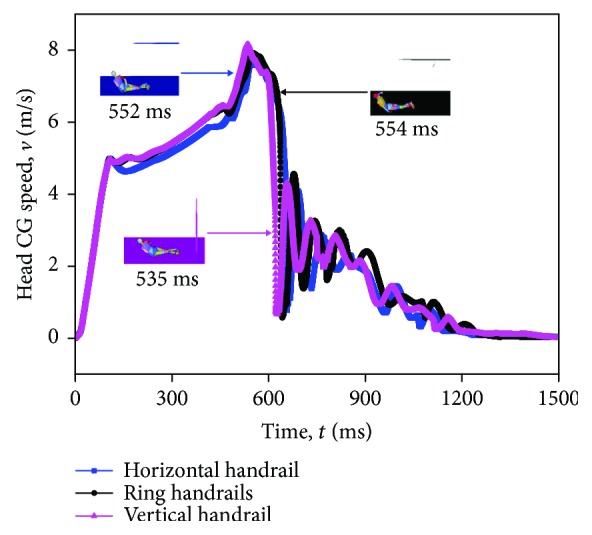
Head CG speed-time history of three passengers with different standing postures in the baseline scenario.

The speed-time history curves of the standing subway passenger head center of gravity in three standing postures are indicated in Figure 10. The general trends of the curves are ascending with some little fluctuations before the peak appears, due to the hand force of the standing subway passenger. The speed of head CG reaches the peak when the abdomen-vehicle contact happens, caused by a sudden drop. Later, there is a wave of the speed after the head-vehicle contact appears. Subsequently, the head center of gravity speed tends to be relatively steady.

### 3.5. The Head Injury Analysis in FE Model

**Figure 11:**
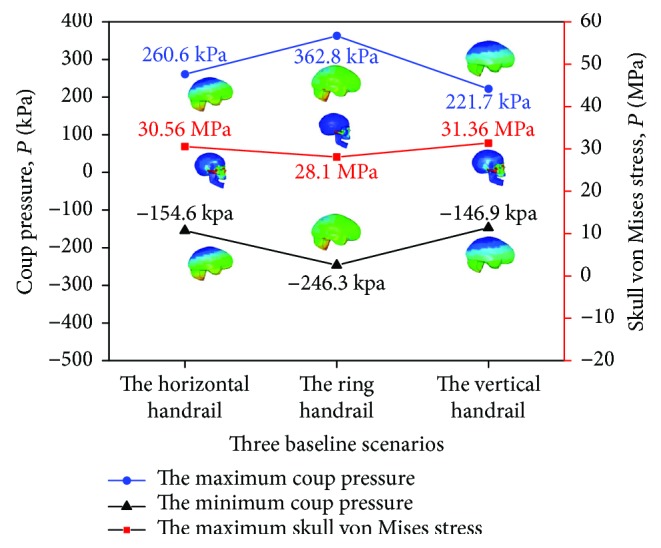
Relation of three passengers with different standing postures and FE head-ground impact model analysis results in the baseline scenario.

Three baseline scenarios are selected in three different standing passenger postures to assess the head injury by FE head-ground impact model. Head injury with coup pressure and skull von Mises stress is analyzed and the result is showed in Figure 11. The maximum coup pressure (362.8 kPa) and the minimum coup pressure (−246.3 kPa) happen in the ring handrail scenario. And the maximum skull von Mises stress (31.36 MPa) happens in the vertical handrail scenarios. All the coup pressures are graded distribution. The maximum skull von Mises stress concentrates on the zygoma.

## 4. Discussion

### 4.1. Parametric Study for Various Coefficient Friction

**Figure 12:**
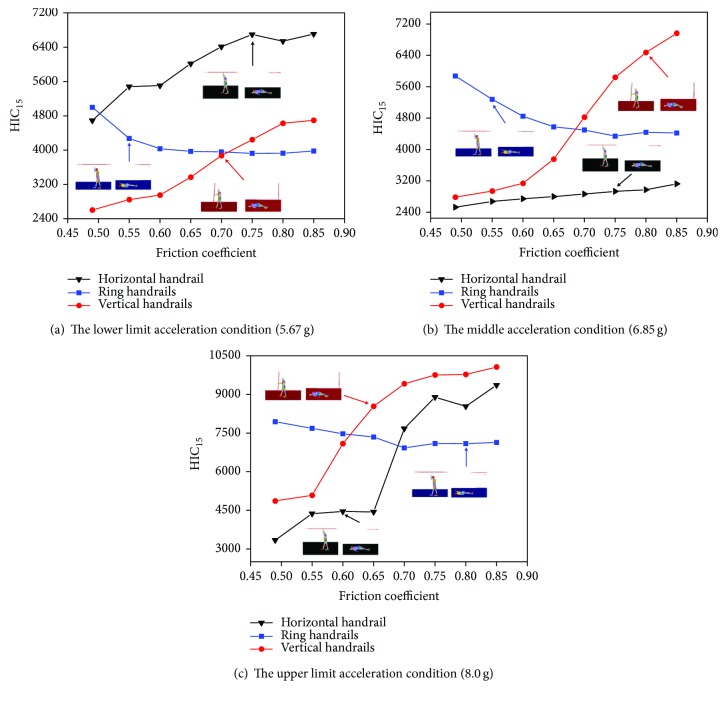
The relation of the coefficient friction and HIC_15_ values of three passengers with different standing postures in three collision acceleration conditions.

The coefficient friction between the passenger and the ground has a significant effect during collisions [28]. In Figure 12, the relation between the head injury HIC_15_ value and the coefficient friction is illustrated. Different impact acceleration conditions are considered: the lower limit acceleration (5.67 g), the middle acceleration (6.85 g), and the upper limit acceleration (8.0 g) in Figures 12(a)–12(c), respectively. The other parameters are kept the same as those in the baseline case. The results show that HIC_15_ of the standing subway passenger in the horizontal handrail and the vertical handrail increases with the increasing of friction coefficient, while HIC_15_ of the ring handrail decreases with the increasing of friction coefficient. This indicates the friction coefficient has a huge effect in different standing postures.

**Figure 13:**
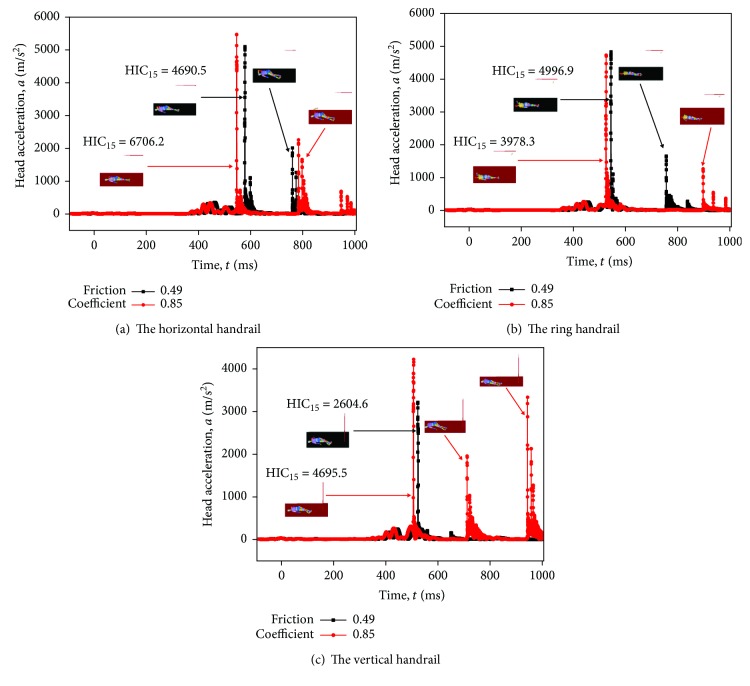
Head acceleration-time history of three passengers with different standing postures in the lower limit acceleration (5.67 g).

The maximum and minimum of HIC_15_ (in Figure 12(a), a baseline scenario) in three postures are employed to investigate how the friction coefficient influences the impact mechanism. As shown in Figures 13(a)–13(c), the time of head-floor contact in three postures is varied. The head-floor contact comes first when the friction coefficient is 0.85, compared with 0.49 in three postures. It can be explained that the time of hand-handrail detachment is different and the greater of the friction coefficient makes people fall down faster during the collisions. These findings can be used by vehicle manufacturers to reduce head injury.

### 4.2. Parametric Study for Various Collision Acceleration

**Figure 14:**
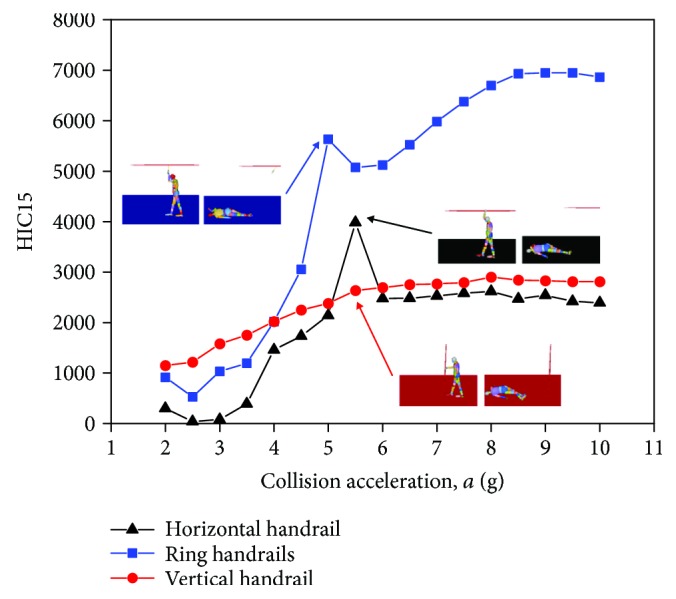
The relation of the collision acceleration condition and HIC_15_ values of three passengers with different standing postures.

It is obvious that the collision condition is a dominant factor. It is necessary to quantify the influence on the head injuries brought by the collision condition. As shown in Figure 14, obviously, the overall curves rise with the increasing of the collision acceleration. The curve changes are not normal when the collision acceleration is less than 3 g. It can be explained that there is no consideration to self-balancing mechanism of the standing subway passenger model.

**Figure 15:**
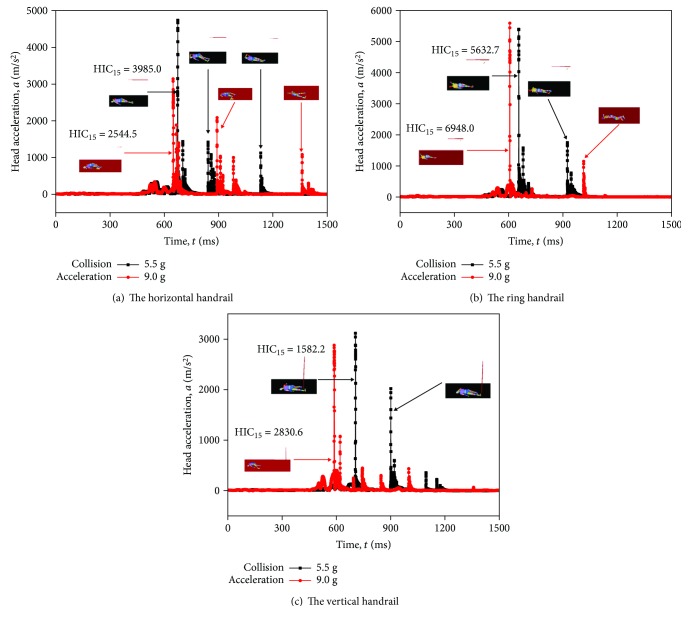
Head acceleration-time history of three passengers with different standing postures.

Compared with HIC_15_ of the three postures, respectively, there is a point of mutation around 5.5 g. HIC_15_ scores are quite big when the collision acceleration is 5.5 g in the horizontal handrail and 5.0 g in the ring handrail. The maximum HIC_15_ of three postures appears in 9.0 g. It can be observed in Figures 15(a)–15(c). Surprisingly, the point of mutation around 5.5 g is close to the lower limit acceleration in AV/ST 9001. These findings have a certain reference for the establishment of collision standards.

### 4.3. Parametric Study for Various Standing Angles

**Figure 16:**
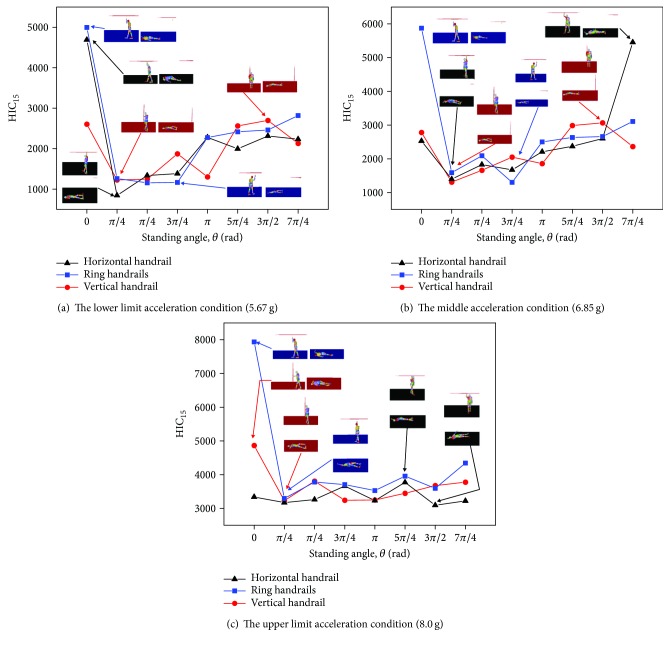
The relation of the standing angle and HIC_15_ values of three passengers with different standing postures in three collision acceleration conditions.

The standing angle is considered as a variable [9]. Figures 16(a)–16(c) show the three postures of the standing subway passenger with different standing angles under the lower limit acceleration (5.67 g), the middle acceleration (6.85 g), and the upper limit acceleration (8.0 g), respectively.

The varying trends of HIC_15_ at various standing angles are somewhat irregular. As shown in Figure 16, for the horizontal handrail of the standing subway passenger, the HIC_15_ is smaller when the standing angle is *π*/4. The HIC_15_ is quite bigger, when the standing angles are 0 and 7*π*/4. For the ring handrail of the standing subway passenger, the HIC_15_ reaches the biggest, when the standing angle is 0. The smallest HIC_15_ happens in the 3*π*/4 or *π*/4. As for the vertical handrail of the standing subway passenger, the standing angle of *π*/4 causes the smallest HIC_15_, while the biggest HIC_15_ happens in 0 and 3*π*/2. In general, the maximum HIC_15_ appears at both ends of the curve. It can be explained by the head-floor contact directly.

**Figure 17:**
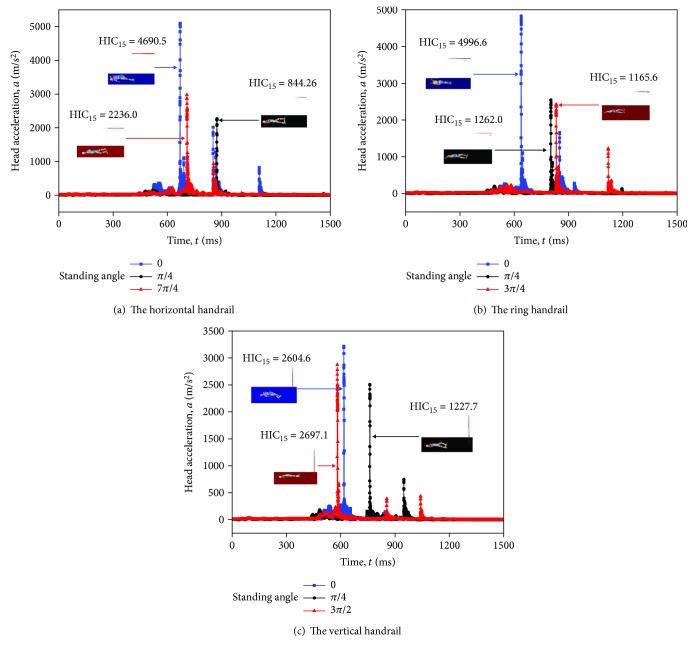
Head acceleration-time history of three passengers with different standing postures in the lower limit acceleration (5.67 g).

To figure out detail contact behaviors in the collision, the head acceleration curves are extracted along with three postures in Figures 17(a)–17(c). It can be observed that the HIC_15_ is higher when the time of head contact becomes earlier. The reason is that the hand plays a protective role, which means hand-floor contact comes first compared with head-floor contact and the HIC_15_ is smaller at the same time. This finding provides some guidance for the standing direction to subway passengers.

### 4.4. Parametric Study for Various Heights

**Figure 18:**
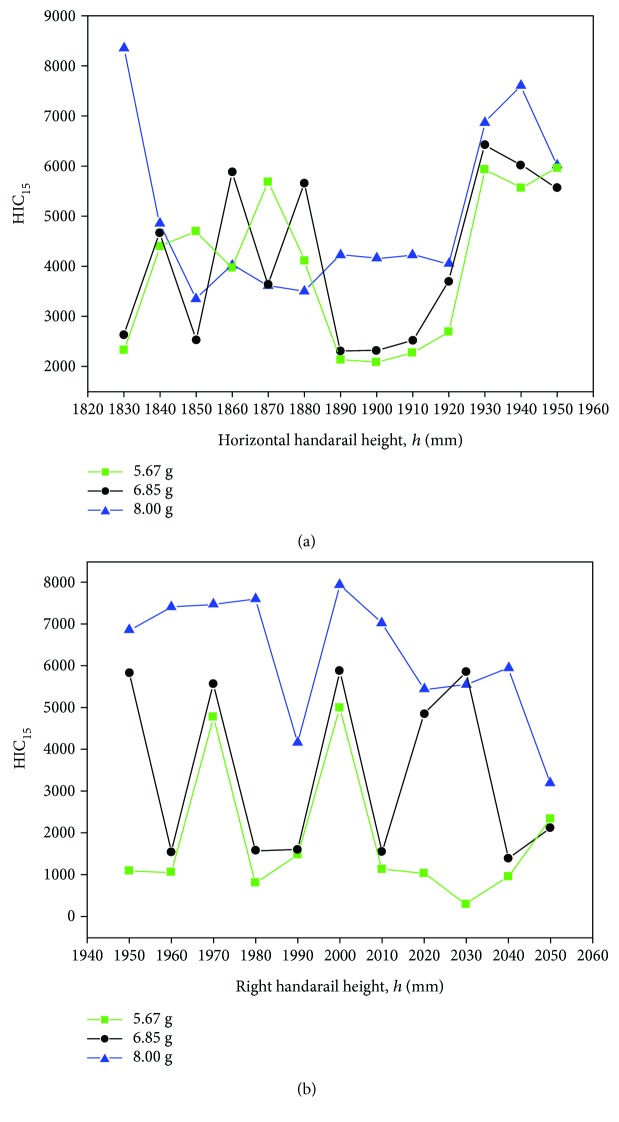
The relation of the height and HIC_15_ values in three acceleration conditions.

Previous research and design indicated that the height of the handrail might bring different consequences to the injuries of the subway passenger during the collisions [10]. It is also interesting to discuss the influence caused by various heights of the handrail in the standing subway passenger head injuries. The standard heights of the horizontal handrail and the ring handrail are 1850 mm and 2000 mm, respectively. Figures 18(a) and 18(b) show the relationship between the HIC_15_ values and heights of the horizontal handrail and the ring handrail in three collision conditions. From the curves, the 6.85 g and 8.00 g scenarios in the ring handrail at 2000 mm height have high injury values but they are much lower at both the 1990 mm and 2010 mm heights. For the horizontal handrail, the standard height 1850 mm is on the middle level. The HIC_15_ values are sensitive to the height of the handrail. This finding is helpful to the handrail design, besides thinking about ergonomics.

## 5. Conclusion

In this paper, numerical collision condition was set up to investigate the head injuries of standing passengers during a crash with three standing postures, that is, the horizontal handrail, the ring handrail, and the vertical handrail. Three baseline scenarios were set with three different standing subway passenger postures. The head finite element model was studied to emphasize the head injury importance in the three baseline scenarios. Then, parametric studies were carried out in the baseline scenarios, such as coefficient friction, collision acceleration, standing angle, and handrail heights.

Based on the analysis, the following changes or suggestions were proposed for the subway and the standing subway passenger. A lower stiffness of the rubber used for the floor and the appropriate handrail height should be considered. Results showed that the bigger acceleration was likely to result in more serious head injuries in the standing subway passenger. Therefore, driver training should be included to brake faster when the collision occurs. The standing subway passenger should be discouraged from standing in a certain angle toward the subway moving direction. According to 195 numerical simulations of this paper (besides 75 numerical simulations in the study for various heights), the number of cases that the horizontal handrail obtains the lowest HIC_15_ values accounts for 61.5% (40 out of 65) of all simulations. The horizontal handrail is safer, compared with the ring handrail and the vertical handrail.

It should be noted that before drawing the final conclusion about the head injuries of standing passengers during a crash, more researches need to be extended in the future, due to the limitations in this paper. Firstly, balance of human body requires closed loop control to generate a complex sway movement using neural signals to activate muscles. We do not take balance loss into consideration. It is inaccurate under the emergency braking conditions. Secondly, head injury is just one of the factors which may result in the standing subway passenger injury. Other lethal injuries are not considered in this paper, such as abdominal injury and serious thoracic trauma. Thirdly, only the standing subway passenger was considered, while the sitting subway passenger was uninformed. Finally, the FE impact simulations with only the FE head model (non-FE human body model) may have some limitations, so a pedestrian FE model (e.g., GHBMC model or THIMS model) will be considered in the future.

## Figures and Tables

**Table 1:** Different parameter ranges in 270 numerical simulations.

Parameter	Range
Coefficient friction	0.49, 0.55, 0.60, 0.65, 0.70, 0.75, 0.80, 0.85
Collision acceleration (g)	2, 2.5, 3.0, 3.5, 4.0, 4.5, 5.0, 5.5, 6.0, 6.5, 7.0, 7.5, 8.0, 8.5, 9.0, 9.5, 10.0
Standing angle (rad)	0, π/4, *π*/2, 3π/4, π, 5π/4, 3π/2, 7π/4
Heights of the horizontal handrail (mm)	1830, 1840, 1850, 1860, 1870, 1880, 1890, 1900, 1910, 1920, 1930, 1940, 1950
Heights of the ring handrail (mm)	1950, 1960, 1970, 1980, 1990, 2000, 2010, 2020, 2030, 2040, 2050
